# Improving the Pipeline for Developing and Testing Pharmacological Treatments in Pregnancy

**DOI:** 10.1371/journal.pmed.1002161

**Published:** 2016-11-01

**Authors:** Lucy C. Chappell, Anna L. David

**Affiliations:** 1 Women's Health Academic Centre, King’s College London, London, United Kingdom; 2 Institute for Women's Health, University College London, London, United Kingdom

## Abstract

In a Perspective, Lucy Chappell and Anna David discuss ways to develop and test pharmacological treatments in pregnancy.

The mismatch between the burden of disease for pregnant women and their infants on the one hand and investment in developing and testing pharmacological treatments on the other continues to be a barrier to successfully reducing morbidity and mortality in this important group. Physiological changes in pregnancy commonly, but not universally, result in increased drug clearance and decreased exposure to total drugs (bound and unbound to plasma proteins) at a given dose. In a systematic review in this week’s *PLOS Medicine*, Shinya Ito and colleagues draw attention to the relative paucity of knowledge about how these pregnancy-induced pharmacokinetic changes impact on clinical outcomes for the mother and fetus after drug treatment [1]. More studies exploring the pharmacodynamic impacts on clinical efficacy in relation to these pharmacokinetic changes are urgently needed to inform drug dosing in pregnancy. This would ensure that pregnant women receive adequate concentrations of an active drug whilst avoiding unnecessary and excessive exposure of their embryo or fetus to potentially teratogenic compounds.

Many drugs used in pregnancy are borrowed or repurposed from mainstream therapeutics, with comprehensive evidence of efficacy and safety in pregnant women often lacking despite awareness of the pharmacokinetic changes described by Ito and colleagues. Despite the well-publicised accounts of teratogenicity—including limb deformities in the fetus—associated with thalidomide (used for hyperemesis) [2] and of the association of diethylstilbestrol (used to prevent early miscarriage) with clear cell adenocarcinoma of the vagina and cervix in daughters of exposed pregnant women [3], the majority of drugs prescribed in pregnancy are unlicensed for use in this setting (i.e., used off-label), with much less safety testing than that undertaken for original marketing authorisation. The strengthening of pharmaceutical regulatory procedures after thalidomide means that it is hardly surprising that, for these and other reasons, the pharmaceutical industry has invested far less in development of new drugs in pregnancy compared to other areas, where the safety hurdles may be less overwhelming and the duration of treatment longer than the time-limited use in pregnancy. This was enumerated in 2007 through a review of an industry database reporting that only 17 drugs were under active development for maternal health indications; this was a fraction of the number for cardiovascular health (660 drugs) and fewer than for a single rare disease such as amyotrophic lateral sclerosis (34 drugs) [4].

Estimates of medication use in pregnancy using interviews or population-based cohorts reveal high usage: in one longitudinal study from the United States, 96% of the pregnant women took prescription medications, 93% self-medicated with over-the-counter medications, and 45% used herbal medications [5]; other studies show similar use and reflect the increasing comorbidities of pregnant women (e.g., diabetes, hypertension, asthma, depression) for which medication is commonly prescribed. A review of inpatient antenatal prescriptions in a United Kingdom maternity unit reported that of 235 drugs prescribed, only 16% were licensed for use in pregnancy; one quarter (24%) of drugs were prescribed off-label but were considered safe by the manufacturers (e.g., erythromycin, prochlorperazine); for over half (58%) of these 235 drugs, the manufacturer advised caution or contraindication. [6] As the majority of drugs used by pregnant women are not specifically licensed for use in pregnancy, detailed pharmacokinetic and pharmacodynamic studies have often not been undertaken to evaluate efficacy (or safety) in this distinct physiological setting. Ito and colleagues show that associated alterations in clinical responses and outcomes, or lack thereof, remain largely unknown. Drugs with minimal side effects, when taken by a nonpregnant adult, may have specific fetotoxic effects when taken during pregnancy, particularly in the first trimester. For example, many antiepileptic drugs are associated with fetal neural tube defects and cardiac abnormalities, most likely because of disruption of folic acid metabolism. However, stopping such drugs may confer risks of uncontrolled maternal seizure activity, which in itself may be life-threatening to mother and fetus. For some drugs, the pattern of potential teratogenicity is not always predictable from animal studies and may only reveal itself once the drug is taken by pregnant women. Distinct differences in placentation (the human placenta contains one layer of cells between maternal and fetal blood, whereas most other animal species have multiple layers), placental metabolism, clearance, and availability of active drug may be responsible. This reinforces the need for preauthorisation research in pregnant women, but undertaking reproductive toxicology studies, to the extent that will satisfy the regulatory agencies, is time-consuming and expensive.

The difficulty of adequate testing is compounded by the paucity of suitable animal models for several pregnancy-specific diseases such as pre-eclampsia and preterm labour, both of which have heterogeneous pathophysiological pathways in the human and no clear animal equivalent. The development of novel drugs for these indications—such as atosiban, a competitive oxytocin receptor antagonist for the treatment of threatened preterm labour—has been beleaguered by controversy, such as uncertainty over appropriate clinical endpoints in trials (i.e., hard neonatal outcomes rather than prolongation of gestational age) [7] and licensing in some geographical areas (e.g., Europe) but not others (e.g., in the US) because of differences in interpretation of trial data. Teratology information services [8] play a key role in screening for potential new human teratogens, particularly in gathering information about newly marketed medications. We find ourselves, however, in the relatively unsatisfactory position that, currently, the use of new drugs in pregnant women creeps into clinical practice and is evaluated via postmarketing surveillance or registry studies, if at all [9], with pregnancy even being a contraindication to participation in many phase IV studies.

Two strands of development are now urgently needed: a greater understanding about the impact in pregnancy of drugs commonly used for coexistent medical conditions—including infections (e.g., antibiotics, antivirals, antimalarials) and chronic diseases (e.g., hypertension, epilepsy, asthma, diabetes, rheumatological diseases)—and a coordinated strategy to invest in the development of drugs for pregnancy-specific conditions such as pre-eclampsia, fetal growth restriction, and preterm labour, where disease-modifying pharmacological treatments could have a major impact on ameliorating short- and long-term adverse outcomes for mother and baby. The importance of the intrauterine environment on adult health is well known [10], but estimating the cost-effectiveness of drugs given in pregnancy that ameliorate adult disease is challenging. Databases for the “repurposing” of drugs with licenses for use in obstetrics are emerging, but further work is needed to understand the potential market revenues for obstetric therapeutics and to encourage investment from the pharmaceutical industry.

Clinical trials in pregnant women can be challenging and require collaborative efforts across clinical and research networks along with the active involvement of the pharmaceutical industry. In parallel, there is a need for ongoing postmarketing surveillance (with good linkage to paediatric developmental outcome data) of drugs licensed for other medical conditions that are used commonly but without adequate knowledge on dosing (e.g., antibiotics, or low-molecular-weight heparin for thromboprophylaxis) or used rarely but where there are compelling reasons to continue treatment (e.g., biologics). New European Union regulations introduced in 2007 mandated the requirement for a Paediatric Investigational Plan for all applications for marketing authorisation for new medicines (unless the medicine is exempt because of a deferral or waiver) [11]. Such an obligation could be proposed for pregnancy at a legislative level to increase the formal testing of medicines on women who are pregnant. This commitment would require considerable financial resources and willingness for change, but the ongoing economic, health, and psychosocial costs associated with diseases in pregnancy necessitate a transformational approach. The recent changes to the US Food and Drug Administration rules on labelling of medications in pregnancy and lactation, which replaces the previous letter categories (A, B, C, D, X) with an informative narrative description of potential benefits and risks and establishes links to pregnancy exposure registries, are to be welcomed [12]. We and others [13] now propose that the following avenues (Box 1) need to be explored by all those involved with medical care for women of reproductive age and the lifelong consequences of pregnancy-associated diseases to ensure that this gap in our research and clinical knowledge is addressed.

**Box 1. Strategies to Improve the Development and Testing of Pharmacological Treatments in Pregnancy**

- Development of appropriate models in which to test placental transfer and evaluate teratogenesis
- Funding initiatives for pharmacokinetic and pharmacodynamic studies of existing drugs, particularly those unlicensed but commonly used
- Consideration of novel approaches to overcome current ethical, regulatory, and deliverability hurdles for clinical trials in pregnancy
- Institution of funded registries (with obligatory reporting), particularly for drugs for which there is overwhelming clinical need but limited safety data (e.g., new antiepileptic drugs; biologics), with short- and long-term maternal and child neurodevelopmental follow-up
- Establishment of a Maternity Investigational Plan to match that introduced across Europe for medicines in children
- Involvement of pregnant women and their families in all such initiatives such that ethical risks and benefits are explored openly and transparently.
